# Parasite-Dependent Expansion of TNF Receptor II–Positive Regulatory T Cells with Enhanced Suppressive Activity in Adults with Severe Malaria

**DOI:** 10.1371/journal.ppat.1000402

**Published:** 2009-04-24

**Authors:** Gabriela Minigo, Tonia Woodberry, Kim A. Piera, Ervi Salwati, Emiliana Tjitra, Enny Kenangalem, Ric N. Price, Christian R. Engwerda, Nicholas M. Anstey, Magdalena Plebanski

**Affiliations:** 1 Department of Immunology, Monash University, Alfred Medical Research and Education Precinct, Melbourne, Australia; 2 International Health Division, Menzies School of Health Research (MSHR) and Charles Darwin University, Darwin, Australia; 3 National Institute of Health Research and Development (NIHRD), Ministry of Health, Jakarta, Indonesia; 4 NIHRD-MSHR Collaborative Research Program and District Health Authority, Timika, Papua, Indonesia; 5 Centre for Vaccinology and Tropical Medicine, Nuffield Department of Clinical Medicine, Churchill Hospital, Oxford, United Kingdom; 6 Queensland Institute of Medical Research, Brisbane, Australia; Case Western Reserve University, United States of America

## Abstract

Severe *Plasmodium falciparum* malaria is a major cause of global mortality, yet the immunological factors underlying progression to severe disease remain unclear. CD4^+^CD25^+^ regulatory T cells (Treg cells) are associated with impaired T cell control of *Plasmodium spp* infection. We investigated the relationship between Treg cells, parasite biomass, and *P. falciparum* malaria disease severity in adults living in a malaria-endemic region of Indonesia. CD4^+^CD25^+^Foxp3^+^CD127^lo^ Treg cells were significantly elevated in patients with uncomplicated (UM; n = 17) and severe malaria (SM; n = 16) relative to exposed asymptomatic controls (AC; n = 10). In patients with SM, Treg cell frequency correlated positively with parasitemia (r = 0.79, p = 0.0003) and total parasite biomass (r = 0.87, p<0.001), both major determinants for the development of severe and fatal malaria, and Treg cells were significantly increased in hyperparasitemia. There was a further significant correlation between Treg cell frequency and plasma concentrations of soluble tumor necrosis factor receptor II (TNFRII) in SM. A subset of TNFRII^+^ Treg cells with high expression of Foxp3 was increased in severe relative to uncomplicated malaria. In vitro, *P. falciparum*–infected red blood cells dose dependently induced TNFRII^+^Foxp3^hi^ Treg cells in PBMC from malaria-unexposed donors which showed greater suppressive activity than TNFRII^−^ Treg cells. The selective enrichment of the Treg cell compartment for a maximally suppressive TNFRII^+^Foxp3^hi^ Treg subset in severe malaria provides a potential link between immune suppression, increased parasite biomass, and malaria disease severity. The findings caution against the induction of TNFRII^+^Foxp3^hi^ Treg cells when developing effective malaria vaccines.

## Author Summary

Malaria is a major global health problem responsible for more than 1 million deaths annually. Severity of malaria disease is associated with the inability of host immune cells to efficiently eliminate malaria parasites from the blood. Little is known about immune regulatory factors controlling the onset of severe and potentially fatal malaria. Regulatory T (Treg) cells are a small specialized subset of immune cells that suppress the activation and expansion of effector immune cells which partake in parasite elimination. We investigated the relationship between Treg cells, parasite burden, and disease severity in adult malaria patients with either uncomplicated or severe malaria. We demonstrated that Treg cell frequency was elevated in malaria patients and associated with high parasite burden in severe malaria but not in uncomplicated malaria. Comparison of Treg cell characteristics allowed us to identify a new highly suppressive subset of Treg cells that was elevated in severe malaria patients. Our results indicate that severe malaria is accompanied by the induction of highly suppressive Treg cells that can promote parasite growth and caution against the induction of these Treg cells when developing effective malaria vaccines.

## Introduction

Infection with *Plasmodium falciparum* causes over 1 million deaths each year and an effective malaria vaccine remains elusive. The cellular immune responses controlling parasite replication and disease severity in falciparum malaria are not fully understood. Natural Treg cells characterized by the expression of the transcription factor forkhead box P3 (Foxp3) [1],[2], the IL-2 receptor α chain CD25 [3] and low expression levels of the IL-7 receptor α (CD127) [4],[5], are widely accepted to be a specialized immunosuppressive subpopulation of CD4^+^ T cells [2]. Treg cells suppress cellular immune responses through direct contact with immune effector cells and via the production of regulatory cytokines, including IL-10 [6],[7]. Whilst it is understood that effector T cell responses are important components of host anti-parasitic immunity [8],[9], the role of Treg cells as suppressors of T cell responses in malaria remains unclear. In mouse experimental malaria models, Treg cells have been associated with increased [10],[11] or delayed [12] disease progression. Importantly, in each of these studies, Treg depletion was associated with improved control of parasite growth.

Pre-clinical blood stage *P. falciparum* infection initiated by sporozoite challenge results in an expansion of Treg cell numbers in malaria-naïve, adult volunteers associated with increased parasite replication [13]. Studies of natural infection are limited, but have shown that Treg cell numbers are increased in neonates born to mothers with placental *P. falciparum* malaria [14] and, in a cohort study in a malaria-endemic region of Kenya, higher baseline Treg cell numbers are associated with an increased risk of subsequent development of clinical malaria [15]. Recent studies have demonstrated a functional deficit in Treg cells in Fulani, an ethnic group with lesser parasitization and a lower risk of clinical malaria [16]. Taken together, these studies imply an important role for Treg cells in the development of clinical malaria, with suppression of T cell-mediated control of parasite replication a plausible explanation [13]. The role of Treg cells in modifying disease severity in malaria is currently unknown and their relationship to parasite biomass has not been examined. The latter has fundamental implications for clinical presentation since parasite replication and biomass are major determinants of the development of severe and fatal malaria [17]–[19].

To better understand the role of Treg cells in disease progression/severity and their relationship with parasite biomass, we examined Treg cell frequency, phenotype and function in adults with uncomplicated (UM) or severe falciparum malaria (SM) in a malaria-endemic area of Papua, Indonesia. Our findings indicate that increased Treg cell numbers are strongly associated with peripheral parasitemia and parasite biomass in severe disease, providing a plausible explanation for a key role of Treg cells in parasite replication and disease severity. Furthermore, we observed an altered Treg cell phenotype in severe disease, with the induction of a TNFRII^+^ population recently associated with ‘maximally suppressive’ activity in animal models [20]. The functional consequences of this finding may underlie progression to hyperparasitemia and severe disease.

## Results

### Study cohort

PBMC from 33 adult patients with *P. falciparum* malaria (17 with UM, 16 with SM), and 10 malaria exposed asymptomatic control (AC) residents were studied (Table 1). Peripheral blood parasitemia and total parasite biomass were each significantly higher in SM patients, compared to UM patients (p = 0.004 and 0.002 respectively, Table 1). A Coulter count of absolute lymphocytes in whole blood showed no significant differences between control subjects and the UM or SM patients (Table 1). Flow cytometry analysis also did not demonstrate a significant difference in the percentage of CD4^+^ T cells of lymphocytes among these groups (Table 1).

**Table 1:** Characteristics of subjects providing PBMC for Treg cell evaluation.

	Asymptomatic Malaria-Exposed Control (AC) n = 10	Uncomplicated Malaria (UM) n = 17	Severe Malaria (SM) n = 16
Median age (interquartile range)	25 (19–31)	27 (22–33)	26 (19–39)
Female/Male	1/9	6/11	5/11
Median *P. falciparum*/µL (interquartile range)	31 (0–148)	3,500 (700–17,900)	128,000 (3,500–473,000)
Plasma PfHRP2 levels ng/mL Median (interquartile range)	0.6 (0.6–0.6)^a^	124 (0.9–170)	12,400 (4,800–33,800)
Median lymphocyte count 10^9^/L (interquartile range)^b^	1.75 (0.7–2.1)	1.1 (0.6–1.7)	1.5 (0.8–2.0)
Median % CD4^+^ T cells/lymphocytes (interquartile range)	28 (22–43)	39 (35–52)	31 (20–38)

a In 9/10 subjects, HRP2 levels were below the detection level of 0.06 ng/mL.

b Absolute lymphocyte counts from Coulter data were available for all AC and a subgroup of 11 UM and 11 SM subjects.

### Treg cell frequency is increased in UM and SM

**Figure 1:**
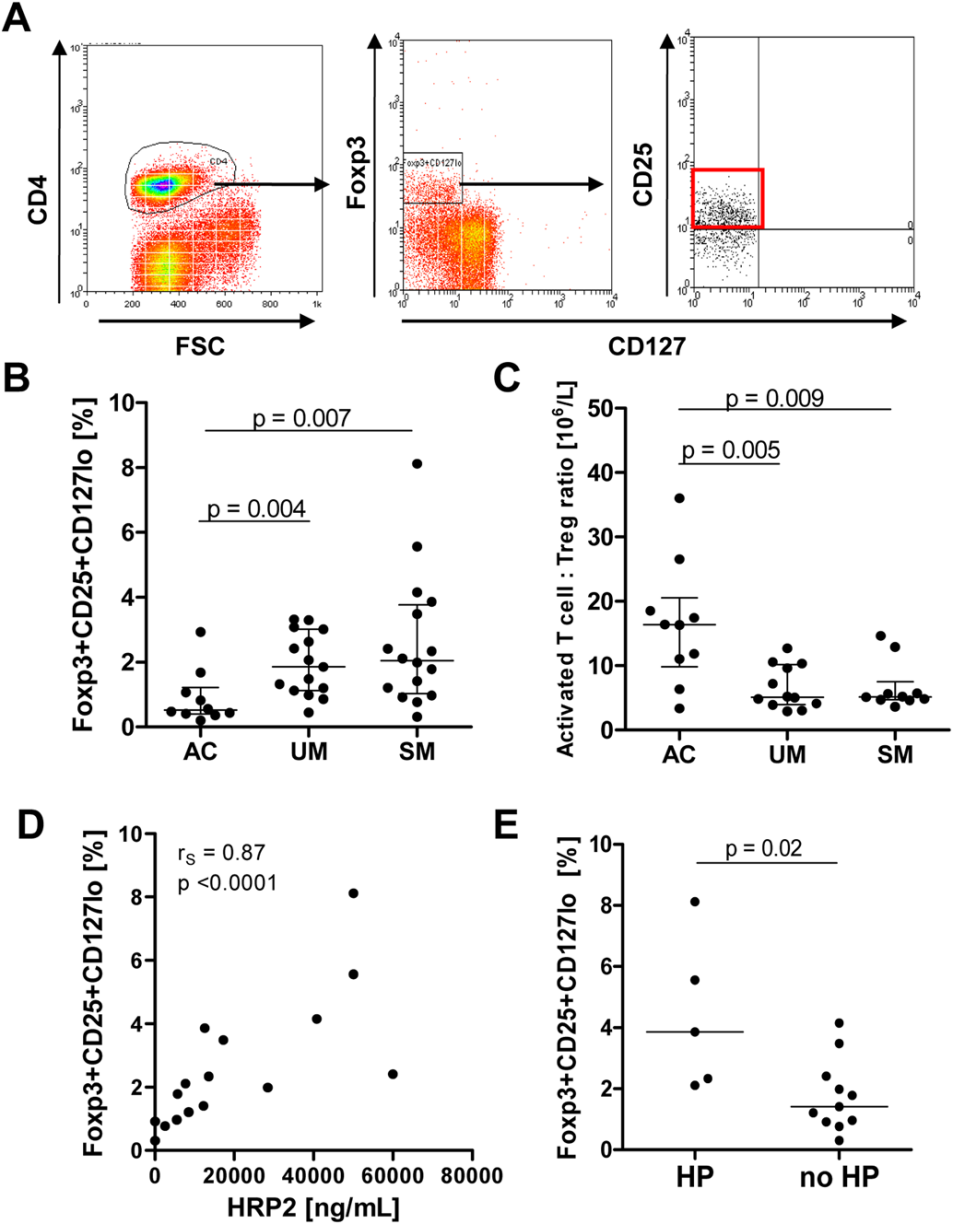
Treg cell analysis. PBMC were stained for the cell surface markers CD4, CD25, and CD127, followed by intracellular staining for Foxp3. (A) Lymphocytes were firstly gated on CD4 expression. All gated CD4 cells expressed CD3 (data not shown). CD4 T cells were then gated on cells expressing Foxp3 with low expression of CD127. Only cells that also expressed CD25 were considered Treg cells. (B) Treg cell frequency was significantly elevated in malaria patients (p = 0.007 by Kruskal-Wallis test, and in post-hoc pairwise comparison with controls, UM: p = 0.004, SM: p = 0.007). Treg cells are shown as the percentage of CD4 T cells. Horizontal lines depict the median with interquartile range. (C) Activated CD4 T cells were defined as CD25+CD4 T cells without co-expression of the Foxp3+CD127^lo^ phenotype. Ratios of activated CD4 T cells: Treg cells are shown for AC, UM, and SM (p = 0.007 by Kruskal-Wallis test, and in post-hoc pairwise comparison with controls, UM: p = 0.005, SM: p = 0.009). Horizontal lines depict the median with interquartile range. (D) Correlation of Treg cell frequency and PfHRP2 plasma levels in SM. (E) In patients with severe malaria, Treg cells were significantly elevated in subjects with hyperparasitemia (HP) compared to SM patients without hyperparasitemia (no HP); p = 0.02; Mann-Whitney U test. AC, malaria-exposed asymptomatic controls; UM, uncomplicated *P. falciparum* malaria; SM, severe *P. falciparum* malaria.

Treg cells were identified by flow cytometry as CD4^+^ T cells expressing Foxp3, CD25, and low levels of CD127 (Figure 1A) and are reported as a percentage of total CD4^+^ T cells. Treg cell frequency was significantly increased in both UM and SM patients (median 1.8% [IQR: 1.1–3.0%] and 2.1% [IQR: 1.0–3.8%], respectively) relative to AC subjects (median 0.52% [IQR: 0.4–1.2%]); Figure 1B, p = 0.006. Absolute Treg cell counts were available for all AC and 11 UM and 11 SM subjects: there were no significant difference in Treg cell frequencies (Figure 1B) or absolute Treg cell numbers between UM and SM patients (median 7.1×10^6^/L [IQR: 4.7–11.9×10^6^/L] and 5.3 [3.3–12.1×10^6^/L], respectively; p = 0.6). Therefore, the number and frequency of Treg cells increased in malaria patients, relative to controls, but did not vary with disease severity.

Activated antigen-experienced effector/memory CD4 T cells were determined as CD4^+^ T cells expressing the activation marker CD25 that did not co-express Foxp3 together with low levels of CD127 [4],[21]. Differences in frequencies and absolute numbers of activated CD4 T cells were not significant between AC (median 8% [IQR: 6.7–11.6%] and 37.7×10^6^/L [IQR: 27.1–52.4×10^6^/L], respectively), UM (median 11.6% [IQR: 9.6–13.7] and 54.1×10^6^/L [IQR: 26.8–75.2×10^6^/L], respectively) and SM groups (median 11.7% [IQR: 7.2–14%] and 38.2×10^6^/L [IQR: 18.7–75.6×10^6^/L], respectively); p = 0.3 and 0.8, respectively. Consequently, significantly lower ratios of activated CD4 T cells : Treg cell numbers were detected in patients with UM and SM compared to the AC subjects (Figure 1C; p = 0.007).

Despite similar Treg cell counts and frequency, effector T cell function, assessed by proliferative responses to the recall antigen PPD, were significantly lower in patients with SM (Median SI: 2 [IQR: 1–5]) compared to patients with UM (Median SI: 11 [IQR: 4–23]); p = 0.009.

### Treg frequency correlates with parasite load in SM but not in UM

In patients with SM, Treg cell frequency was correlated with both peripheral parasitaemia (r_s_ = 0.79, p = 0.0003) and parasite biomass (r_s_ = 0.87, p<0.0001; Figure 1D). In these patients absolute Treg cell numbers were similarly correlated (r_S_ = 0.63 [p = 0.038] and r_S_ = 0.76 [p = 0.01]; respectively). In an *a priori* subgroup analysis, the five 5 SM patients with hyperparasitemia (>100 parasites/1000 RBC) had a median Treg cell frequency of 3.86% [IQR: 2.2–6.8%], significantly higher compared to SM patients without hyperparasitaemia (median 1.4% [IQR: 0.9–2.4%]; p = 0.02); Figure 1E. In contrast, in patients with UM there was no significant correlation between Treg cell number or frequency and peripheral parasitemia or parasite biomass (data not shown).

### Increased surface expression of TNFRII on Treg in SM

**Figure 2:**
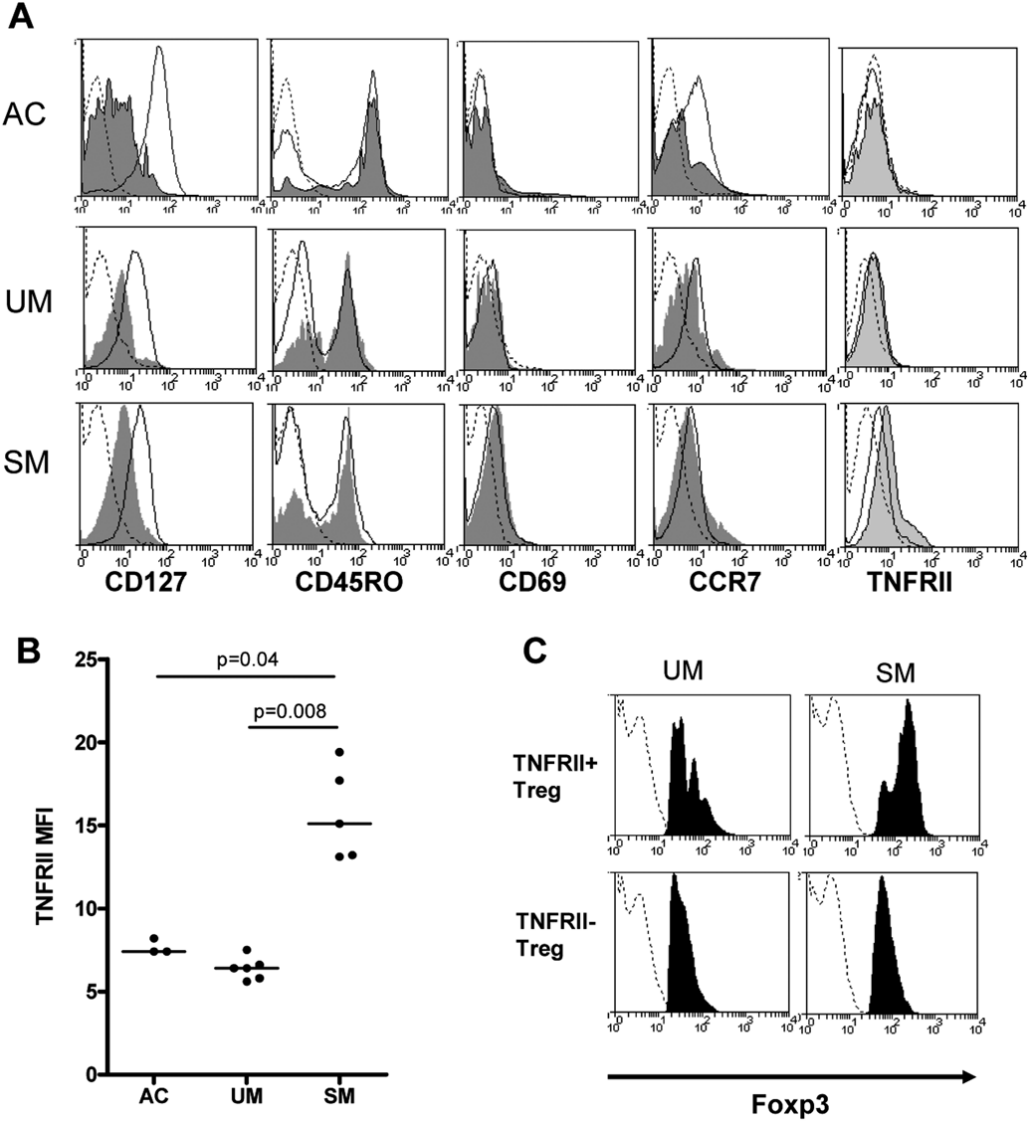
Treg phenotype in UM and SM. PBMC of 3 AC, 6 UM, and 5 SM patients were stained for CD4, CD25, Foxp3, and CD127, or CD45RO, CD69, CCR7, or TNFRII. (A) Mean fluorescent intensities (MFI) are shown for CD4^+^CD25^−^Foxp3^−^ (solid line) and CD4^+^CD25^+^Foxp3^+^ (dark grey) cells or CD4^+^Foxp3^+^CD127^lo^ (light grey) cells. Dotted lines represent the isotype controls. One representative donor is shown, and activated T cells with high expression of CD4 were gated out. (B) Pooled TNFRII MFI (3 AC, 6 UM, 5 SM). Horizontal lines show the median (p = 0.005 by Kruskal-Wallis test, and in post-hoc pairwise comparison of UM and SM: p = 0.008, and between SM and AC: p = 0.04). (C) Foxp3 expression is shown for TNFRII positive Treg cells (top panel) and TNFRII negative Treg cells (bottom panel) for one representative UM and one representative SM sample. The dotted line shows isotype control staining. AC, malaria-exposed asymptomatic controls; UM, uncomplicated *P. falciparum* malaria; SM, severe *P. falciparum* malaria.

Treg cells were assessed further for expression of activation markers. Across the groups CD4^+^CD25^+^Foxp3^+^ T cells expressed low levels of the IL-7 receptor CD127, but not the early activation marker CD69 (Figure 2A). The majority of CD4^+^CD25^+^Foxp3^+^ T cells expressed CD45RO and expression of the lymph node homing receptor CCR7 was low. Since Treg cells have been recently reported to express tumor necrosis factor receptor II (TNFRII), which may contribute to the immunosuppressive functions of these cells [22],[23], TNFRII expression was measured on CD4^+^Foxp3^+^CD127^lo^ T cells (Figure 2A). TNFRII expression was significantly higher on SM CD4^+^Foxp3^+^CD127^lo^ T cells (median MFI: 8.6 [IQR: 7.5–11.5]) compared to UM (median MFI: 5.4 [IQR: 5.3–5.4]; p = 0.008) and AC CD4^+^Foxp3^+^CD127^lo^ T cells (median MFI: 7.4 [IQR: 7.4–8.2]; p = 0.04); Figure 2B. Moreover, a significantly higher frequency of CD4^+^Foxp3^+^CD127^lo^ T cells expressing TNFR2 were found in the SM group (median 19.1% [IQR: 12.7–25.4%]) compared to the UM (median 10.1% [IQR: 4.8–11.3%]) and AC groups (median 9% [IQR: 6.3–10.5%]); p = 0.04.

In both UM and SM patients, Foxp3 expression was higher in TNFRII^+^ Treg cells (combined median MFI: 56 [IQR: 32–103]) compared to TNFRII^−^ Treg cells (combined median MFI: 41 [IQR: 32–63]); p = 0.04; Figure 2C. Together, these data indicate that SM patients have higher numbers of TNFRII^+^ Tregs cells expressing high levels of Foxp3, suggesting that these cells may have greater immunosuppressive capacity.

### TNFRII-expressing Treg cells with enhanced suppressive activity increased after exposure to *P. falciparum* in vitro

**Figure 3:**
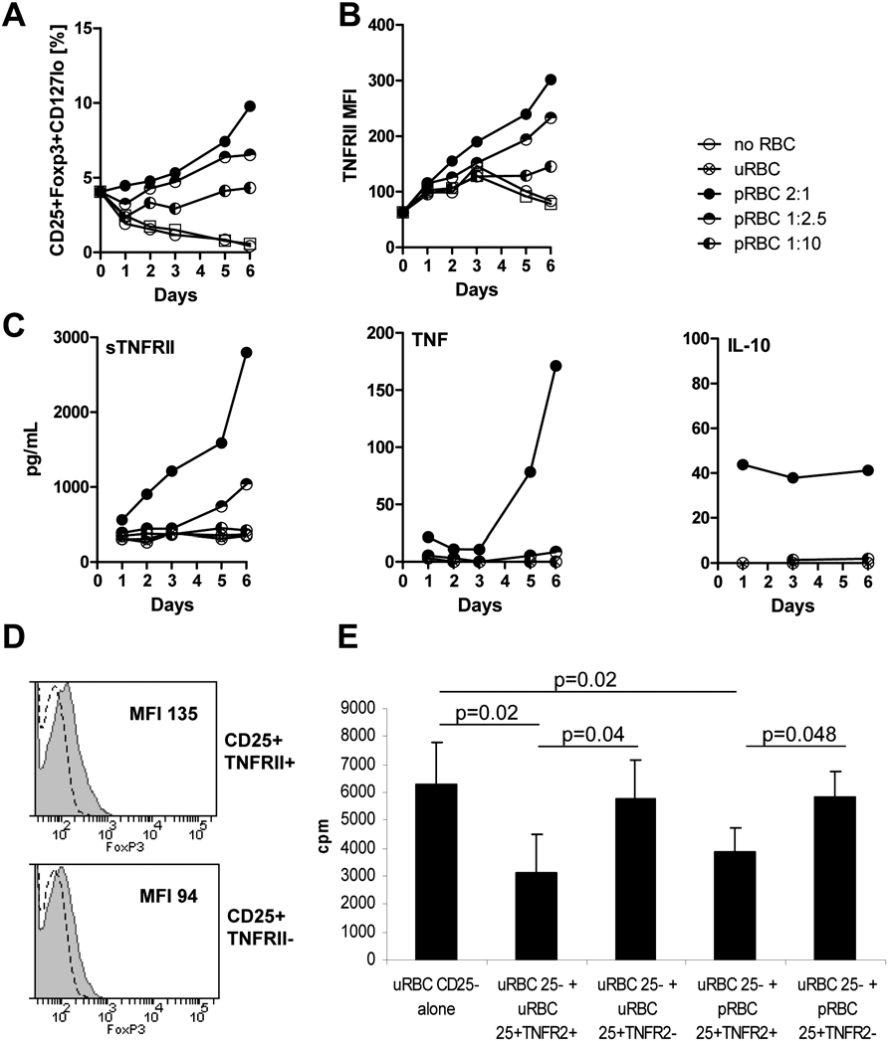
In vitro *P. falciparum* exposure induced Treg cell expansion, TNFRII expression, and enhanced Treg cell activity. PBMC from healthy malaria-unexposed blood donors (n = 4) were cultured overnight in the presence of *P. falciparum*–infected red blood cells (pRBC) at pRBC∶PBMC ratios of 2∶1, 1∶2.5, and 1∶10, or uninfected red blood cells (uRBC) at a uRBC∶PBMC ratio of 2∶1 or without RBC. (A) CD4^+^CD25^+^Foxp3^+^CD127^lo^ Treg cells are represented as percentage of CD4 T cells. (B) TNFRII MFI on CD4^+^ Foxp3^+^CD127^lo^ Treg cells. (C) sTNFRII (left panel), TNF (middle panel), and IL-10 (right panel) concentration in culture supernatants. (D) Following overnight culture with *P. falciparum*–infected red blood cells (pRBC) or uninfected red blood cells (uRBC) at a RBC∶PBMC ratio of 1∶2.5, PBMC were sorted into CD4^+^CD25^−^ responder cells and CD4^+^CD25^+^TNFRII^+^ or CD4^+^CD25^+^TNFRII^−^ Tregs and stained intracellularly for Foxp3. Foxp3 expression is shown for CD4^+^CD25^+^TNFRII^+^ or CD4^+^CD25^+^TNFRII^−^ Treg cells (grey line) and CD4^+^CD25^−^ responder cells (dotted line). (E) Sorted cells were tested for suppressive activity. 10^4^ CD4^+^CD25^−^ responder T cells sorted after uRBC exposure were incubated either alone or with TNFRII^+^ or TNFRII^−^ Tregs after uRBC or pRBC exposure at a 1∶1 ratio in 96 well plates pre-coated with 3 µg/mL anti-CD3 antibody (OKT-3) for 3 days. Sorted monocytes were used as APC. Bars show mean cpm+/−SD of triplicate wells.

The relationship between *P. falciparum* Treg cell expansion and activity was examined using an in vitro cell culture system to control for extraneous variables. PBMC from malaria-unexposed donors were cultured in vitro either alone or in the presence of parasitized red blood cells (pRBC) or uninfected RBC (uRBC) at different ratios. In the absence of RBC or the presence of uRBC, Treg cell frequency declined over 6 days of culture. In contrast, co-culture with pRBC maintained (at lower pRBC ratios) or increased (at higher pRBC ratios) Treg cell frequencies (Figure 3A). This finding was statistically significant using a multilevel regression model (p<0.001) [24]. TNFRII expression by CD4^+^Foxp3^+^CD127^lo^ Treg cells also increased until at least day 6 of culture following pRBC stimulation. Higher levels of Treg cell TNFRII expression were observed in the presence of greater pRBC ratios (Figure 3B). In addition, supernatant concentrations of soluble TNFRII (sTNFRII), an important Treg cell suppressor molecule [22], also increased in the presence of greater pRBC ratios (Figure 3C, left panel). TNF levels were highest in cultures with high parasite ratios. In all donors, TNF levels stayed low over the first three days of culture and increased rapidly from day 4 in cultures with high parasite ratios (Figure 3C, middle panel). Exposure to high, but not low, parasite ratios triggered IL-10 secretion from as early as 24 hours of culture (Figure 3C right panel). Together, these in vitro data may imply a direct association between increased parasite numbers, Treg cell TNFRII expression and cytokine secretion, supporting the in vivo findings suggesting increased parasite exposure may alter Treg cell function.

To assess TNFRII^+^ Treg cell function, CD4^+^CD25^+^ T cells were sorted based on TNFRII expression after 1–2 days of pRBC or uRBC exposure. CD4^+^CD25^+^TNFRII^+^ T cells were found to express higher levels of Foxp3 than CD4^+^CD25^+^TNFRII^−^ T cells (Figure 3D), consistent with our earlier observations on TNFRII^+^ and TNFRII^−^ Treg cells from malaria-exposed individuals (Figure 2C). The suppressive capacity of CD4^+^CD25^+^TNFRII^+^ and CD4^+^CD25^+^TNFRII^−^ T cells was assessed in a standard T cell suppression assay [3] using CD4^+^CD25^−^ responder T cells. Sorted CD4^+^CD25^+^TNFRII^+^ and CD4^+^CD25^+^TNFRII^−^ T cells were added separately to the responder T cells at a Treg : responder T cell ratio of 1∶1. To exclude parasite-mediated changes to the capacities of the responder T cells to proliferate, CD4^+^CD25^−^ responder T cells and antigen presenting cells were isolated from PBMC cultured in the absence of parasite (uRBC co-cultures). Only TNFRII^+^ CD4^+^ CD25^+^ T cells (isolated following PBMC incubation with either uRBC or pRBC) suppressed T cell proliferation following anti-CD3 stimulation (Figure 3E) in 2/3 donors. Responder T cell proliferation was significantly decreased in the presence of CD4^+^CD25^+^TNFRII^+^ T cells compared to responder T cell cultures in the absence of Treg cells (p = 0.02; Figure 3E) and compared to responder T cell proliferation in the presence of CD4^+^CD25^+^TNFRII^−^ T cells (p = 0.04; Figure 3E). These data suggest that TNFR2 expression is a marker of functional Treg cells with suppressive capacity. Our data furthermore indicate that TNFR2^+^Foxp3^hi^ are functional regardless of parasite exposure.

### TGFβ and disease severity

Because TGFβ induces Foxp3 expression [20], we examined whether the Treg cell frequency was associated with plasma TGFβ concentrations. Platelet free plasma was available from a subset of 13 patients (5 AC, 3 UM and 5 SM). In this small subset, no significant relationship was found between TGFβ levels and Treg cells frequency. To clarify this relationship, total TGFβ was therefore measured in archived platelet-free citrated plasma drawn from separate groups of previously studied SM, UM and AC patients from the same population, enrolled using the same study criteria. The levels of TGFβ were elevated in both UM (median 5063 pg/mL; IQR: 3938–5647 pg/mL; n = 20) and SM (median 4178 pg/mL; IQR: 3367–5609 pg/mL; n = 18) patients, relative to controls (median 3213 pg/mL; IQR: 1400–4552 pg/mL; n = 20; p = 0.02). There was no significant difference in TGFβ levels between UM and SM patients and no significant correlation between TGFβ levels, blood parasitemia or parasite biomass (data not shown). These data do not support TGFβ as a Treg cell-derived factor contributing to enhanced parasite burden in SM patients.

### Increased Treg cell numbers correlate with plasma IL-10 and sTNFRII levels

IL-10 plasma levels were significantly increased in patients with UM (median 58.3 pg/mL; IQR: 18.4–117.4 pg/mL; n = 13) and patients with SM (median 203.1 pg/mL; IQR: 61.9–636.3 pg/mL; n = 13) compared to asymptomatic control subjects (median 3.2 pg/mL; IQR: 0.4–9.3 pg/mL; n = 8; p = 0.002 and p = 0.002, respectively). The difference in the plasma levels of IL-10 between patients with UM and SM was not significant. In each of the UM and SM groups, plasma IL-10 correlated positively with Treg cell frequency (r_s_ = 0.66; p = 0.03 and r_s_ = 0.66; p = 0.03, respectively).

**Figure 4:**
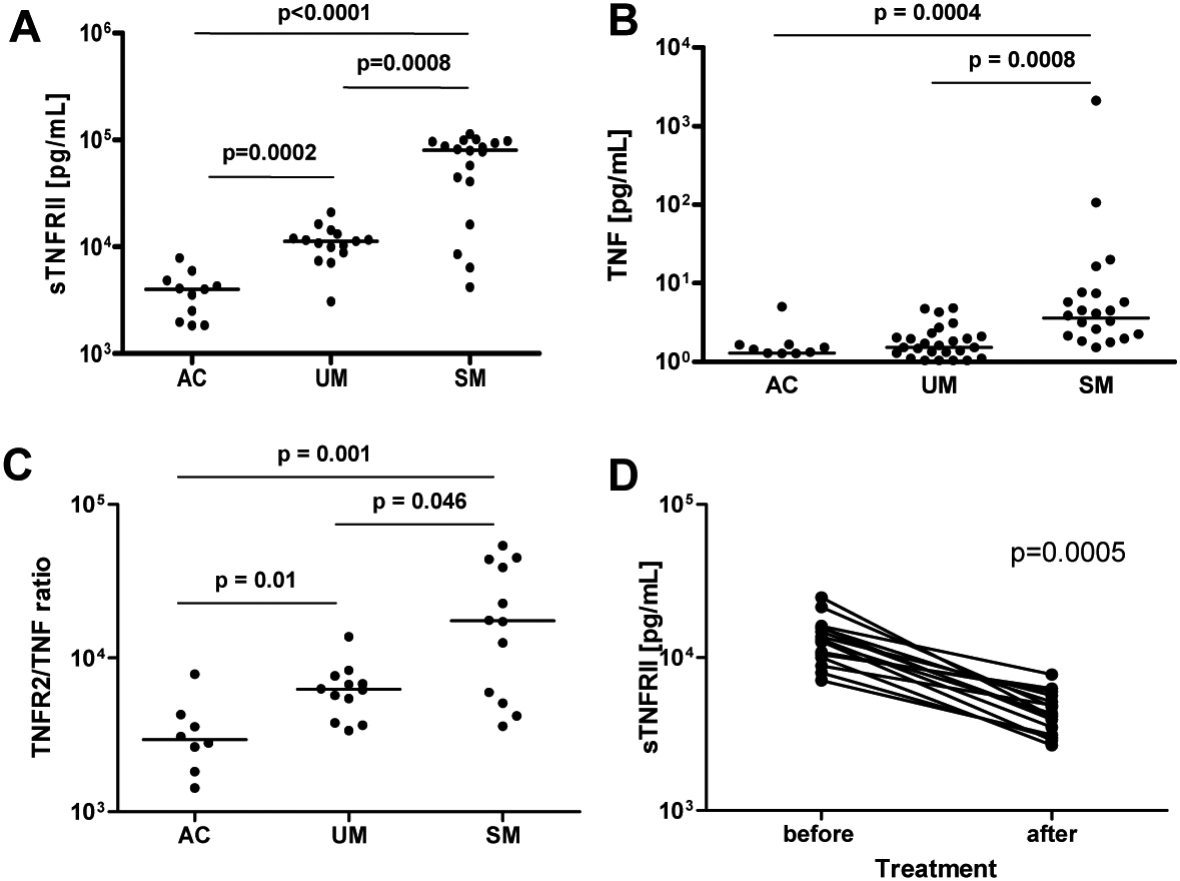
Elevated soluble TNFRII in malaria is associated with disease severity. (A) Plasma levels of soluble TNFRII were elevated in malaria with significant differences between AC, UM, and SM patients. (B) Plasma TNF levels in AC, UM, and SM patients. (C) The ratio of plasma levels of sTNFRII to TNF in AC, UM, and SM patients. (D) In UM, soluble TNFRII levels decreased in convalescence (p = 0.0005; Wilcoxon-rank test). AC, malaria-exposed asymptomatic controls; UM, uncomplicated *P. falciparum* malaria; SM, Severe *P. falciparum* malaria.

sTNFRII plasma levels were significantly associated with malaria disease severity (Figure 4A, p = 0.0008). There was a significant correlation between Treg cell frequency and plasma sTNFRII in SM (r_s_ = 0.59; p = 0.017), but not UM patients. sTNFRII levels were highly correlated with both peripheral parasitemia and parasite biomass in SM (r_s_ = 0.67; p = 0.004 and r_s_ = 0.72, p = 0.004; respectively), but not in UM patients. Plasma concentrations of total TNF (Figure 4B), as well as the sTNFRII∶TNF ratio, were significantly increased in association with disease severity (Figure 4C), suggesting that a greater proportion of total TNF is bound by sTNFRII in severe malaria, potentially reducing TNF bioavailability.

### Treg cell frequency and sTNFRII return to base levels after parasite clearance

Convalescent samples were available in eight patients with uncomplicated malaria 7–28 days after drug treatment. Of these, six (75%) showed reduced Treg cell frequencies 7–28 days after parasite clearance (median 0.9%, IQR: 0.7–1.1%) compared to a median of 1.7% [IQR: 1.0–2.9%] prior to treatment (p = 0.09), falling within the range of Treg cell frequencies in AC subjects (median 0.52% [IQR: 0.4–1.2%]). Plasma, sTNFRII levels were also decreased in convalescent plasma (p = 0.0005; Figure 4D). These data indicate that the malaria-associated increases in sTNFRII levels and Treg cell frequencies are transient and fall following effective anti-parasitic drug treatment.

## Discussion

We report increased CD4^+^CD25^+^Foxp3^+^CD127^lo^ Treg cell frequency in adult patients with uncomplicated and severe *P. falciparum* malaria. While there was no difference in the frequency or absolute counts of Treg cells between UM and SM patients, patients with SM had significantly more TNFRII^+^ Treg cells with elevated Foxp3 expression. Because Foxp3 expression is directly correlated with Treg cell suppressor activity [25], our data suggest that Treg cell activity is increased in severe falciparum malaria. Indeed, PBMC from patients with SM showed poor antigen-specific proliferation relative to PBMC from patients with UM. Although the reasons for impaired immune responses in severe disease may be multi-factorial, this finding is consistent with a greater immunosuppressive capacity of TNFRII^+^ Treg cells in patients with SM. Our in vitro data on PBMC from malaria-unexposed donors further support that TNFRII^+^ Treg cells exert greater suppressive activity relative to TNFRII^−^, which is in accordance with recent findings in mice [20]. Increased TNFRII^+^ Treg cells were found after in vitro exposure of previously malaria-unexposed PBMC to high ratios of pRBC compared to low ratios, suggesting a parasitemia threshold has to be overcome for their induction. While the in vitro model may not necessarily reflect the in vivo response, the in vitro data reflect our observations of increased levels of this suppressive TNFRII^+^Foxp3^hi^ Treg subset in patients with severe malaria who had significantly higher parasitemia and parasite biomass relative to patients with uncomplicated malaria. Due to the cross-sectional nature of this study, which is inherent to human studies of severe malaria, we cannot determine whether the increase in TNFRII^+^ Treg cells in severe malaria is a cause or consequence of progression from uncomplicated to severe disease.

In vitro assays were performed using the *P. falciparum* lab strain 3D7 and while we cannot exclude different reactivity in malaria-exposed subjects it is unlikely that stimulation of PBMC from malaria-unexposed Australian blood bank donors resulted in antigen-specific recall of memory T cell responses to epitopes within the 3D7 strain. Although suppressor function requires Treg cell activation via their T cell receptor, their suppressive capacity is not antigen specific and can promote generalized immune suppression [26]. Furthermore, TNFRII^+^ Treg cells were functional following exposure to parasitized or unparasitized RBC suggesting that the appearance of TNFRII^+^Foxp3^hi^ Treg cells was unlikely to have been promoted by mitogenic molecules from parasite extracts.

The increase in Treg cells in UM and SM indicates that the induction of Treg cells in pre-clinical infection [13], continues into clinical disease. This finding is further supported by the fall in Treg cell frequency and activity following parasite clearance with drug treatment in patients with UM. Importantly, our findings highlight that increased Treg cell numbers are strongly associated with peripheral parasitemia and parasite biomass in severe disease, providing a plausible explanation for a key role of Treg cells in parasite replication and disease severity. While Treg cells may ameliorate potentially deleterious immune responses [11] or endothelial cell activation [27] in severe malaria, our results suggest that in severe disease this may occur at the cost of impairing effector T cell-mediated constraint of parasite growth. Because parasite replication and biomass are major determinants of severe and fatal malaria [17]–[19], Treg cells may thus exacerbate disease severity in SM.

In contrast to previous studies showing impaired TGFβ in uncomplicated and severe malaria in children [28], plasma levels of TGFβ in adults were significantly higher in both uncomplicated and severe malaria. This pattern of TGFβ elevation parallels the pattern seen with elevated Treg cells in uncomplicated and severe disease and is consistent with the known effects of TGFβ in inducing Foxp3 and the Treg phenotype [29]. A limitation of our study is our inability to measure TGFβ in all subjects in whom Treg cells data were also available. Nevertheless, we speculate that differences in the pattern of TGFβ production in uncomplicated and severe disease between adults and children may be associated with differences in Treg responses between these two age groups. As such, our results in adults may not be generalizable to severe malaria in paediatric age groups in whom immune responses and disease phenotypes differ [30].

The anti-inflammatory cytokine IL-10 has been suggested to inhibit pro-inflammatory immune responses to malarial antigen [31]. As found previously [32]–[34], we observed increased plasma levels of IL-10 in adults with both UM and SM. The correlation between plasma IL-10 levels and Treg cell frequency observed in both UM and SM patients is consistent with the known association between IL-10 and Treg cell activity [6],[7]. Although this association does not infer causality, our in vitro data shows that IL-10 is detectable as early as 24 hours following PBMC culture in the presence of high concentrations of pRBC as also found by others [35]. These data lead to the speculation that IL-10 secretion may precede and contribute to the relative increase in Treg cells observed in vitro and ex vivo. Indeed, expansion of Foxp3^hi^Treg cells in vitro can be partially inhibited using anti-IL-10R blocking antibody (Anja Scholzen, personal communication).

Another cytokine recently suggested to expand murine Treg cells in vitro is TNF [36]. In our in vitro cultures, we did not observe an increase in TNF levels until day 4 of culture. Even after day 4 TNF concentrations remained in the pg/mL range, while expansion of murine Treg cells required TNF concentrations in the ng/mL [36]. For these reasons we think it is unlikely that the relative increase in Treg cells following exposure to high pRBC ratios is TNF-mediated. Furthermore, although plasma TNF concentrations were significantly elevated in adults with severe *P. falciparum* malaria, these remain in the low pg/mL range as also reported by others [19],[32].

Recently, the ability of Treg cells to shed large amounts of TNFRII has been identified as a novel mechanism by which Tregs cells can inhibit the effects of TNF [22]. TNF has been associated with accelerated parasite clearance in falciparum malaria [37] and clinical use of TNF inhibitors increases the risk of hyperparasitemia following malaria infection [38]. At low concentrations, soluble TNFRs stabilize TNF and prolong its half-life [39], however at high concentrations the soluble receptors compete for TNF binding with cellular receptors, particularly membrane bound TNF [40], thereby inhibiting TNF bioactivity [41]. Although TNFRII shedding by Treg cells may represent a further counter-regulatory mechanism to suppress potentially deleterious TNF-mediated inflammatory responses induced after rupture of parasitized red blood cells [19],[42], the high concentrations of TNFRII associated with Tregs in SM may inhibit TNF bioactivity and contribute to impaired control of parasite growth and progression to severe disease.

In conclusion our findings support a role for Treg cells in malaria disease progression and parasite growth during clinical malaria and furthermore indicate that *P. falciparum* can expand a potent TNFRII^+^Foxp3^hi^ suppressor Treg cell subset that is associated with, and may contribute to severe malaria and hyperparasitemia in adults. Future studies are indicated in other human populations and age groups assessing the role of this maximally suppressive Treg subpopulation in susceptibility to severe malaria and other inflammatory diseases. Furthermore, differences among candidate malaria vaccines and human populations in the function and phenotype of vaccine-induced Tregs may influence efficacy in protecting against uncomplicated and severe malaria.

## Materials and Methods

### Study site

The study was carried out in the southern lowlands of Papua, Indonesia, where malaria transmission is perennial but unstable with an annual incidence estimated to be 885 per 1000 person years with both *P. falciparum* and *P. vivax* prevalent [43]. Due to economic immigration of largely non-immune residents symptomatic malaria and severe disease is present in all age groups [43].

### Study cohort

Thirty-three malaria-exposed adults participated in this study following informed written consent. Three groups were studied: i) asymptomatic malaria-exposed controls (AC), resident in Timika district for at least two years, with no fever or symptoms of malaria within the preceding two weeks, with or without asymptomatic parasitemia (<1000 parasites/ul); ii) patients with acute uncomplicated falciparum malaria (UM) presenting with *P. falciparum* parasitemia and fever or history of fever within 48 hours and no alternative cause identified [44],[45] iii) patients with *P. falciparum* and ≥1 modified WHO criteria of severe malaria (SM): acute renal failure (creatinine >265 umol/L), or hyperbilirubinemia with renal impairment (creatinine >130 umol/L) and/or parasitemia of >100,000 parasites/uL, or blackwater fever, or hyperparasitemia (>10% parasitised red cells), or cerebral malaria (Glasgow coma score <11), or hypoglycemia [46]. In the SM cohort, 4 patients (25%) had cerebral malaria, 5 (29%) hyperparasitemia, 12 (75%) renal failure, 5 (29%) hyperbilirubinemia and 10 patients (59%) had more than one WHO criterion for severe disease. Peripheral blood mononuclear cells (PBMC) and plasma were cryopreserved and the mean PBMC viability upon thaw was ≥81% (IQR 73–91%) for all subject groups. In the UM group follow-up samples were collected 7 and 28 days after antimalarial treatment [44],[45]. PBMC from Australian Blood Bank donors were used for in vitro assays.

The study was approved by the Ethics Committees of the National Institute of Health Research and Development, Ministry of Health, Jakarta, Indonesia, Menzies School of Health Research, Darwin, Australia and the Australian Red Cross Blood Service.

### Flow cytometric analysis

400 000 PBMC in PBS 2% fetal calf serum were stained with anti- CD3 (HIT3a), CD4 (RPA-T4), CD25 (M-A251), CD127 (hIL-7R-M21), CD45RO (UCHL1), CD69 (FN50), CCR7 (3D12) all BD Biosciences, USA, TNFRII (22235.311, R&D Systems) antibodies or isotype controls conjugated to fluorescein isothiocyanate (FITC), phycoerythrin (PE) or peridinin-chlorophyll-a protein (PerCP). For Foxp3 detection, cells were resuspended in fix/perm buffer (ebioscience, USA), stained with Foxp3 antibody (clone PCH101, ebioscience, USA) conjugated to allophycocyanin (APC), washed, fixed with 1% paraformaldehyde (Sigma, Australia) and acquired using a Becton Dickinson FACScalibur with CellQuest software. FACS data was analyzed using Weasel software (WEHI, Australia).

### Parasite biomass

Total body parasite biomass was quantified by detecting plasma concentration of *P. falciparum* Histidine Rich Protein 2 (PfHRP2) using ELISA as described [18].

### *P. falciparum* trophozoite stimulation of non-malaria–exposed PBMCs and suppression assays

*P. falciparum* 3D7 was maintained in RPMI-1640 medium (JRH, USA) supplemented with 1 mM glutamine, 11 mM glucose, 25 mM HEPES, 0.2% (w/v) sodium bicarbonate, 200 µM hypoxanthine, 40 µg/ml gentamycin (all Sigma-Aldrich, USA), and 0.5% (w/v) AlbuMAX II (Invitrogen, USA) in the presence of O^+^ erythrocytes at 37°C in an atmosphere of 5% CO_2_, 5% O_2_ and 90% N_2_. Trophozoites were isolated on a Percoll (Amersham Biosciences, Sweden) gradient with a yield of >95%. PBMCs collected from healthy Australian donors with no previous malaria exposure, were exposed in culture media to differing ratios of percoll-enriched *P. falciparum* 3D7 trophozite infected RBC (pRBC) or uninfected (uRBC) and cultured for 1–6 days. Culture supernatant was collected for cytokine analysis (below) and cells were analyzed using flow cytometry or sorted for suppression assays. For cell sorting, PBMC were stained with anti-CD4, CD25 and TNFRII monoclonal antibodies. CD4^+^ populations were sorted using the FASCAria (BD Biosciences) and CD25^−^, CD25^+^TNFRII^+^ and CD25^+^TNFRII^−^ cells collected at average purities of 98% (CD25^−^), 61% (CD25^+^TNFRII^+^) and 89% (CD25^+^TNFRII^−^). Suppression assays were performed as previously described [3]. Briefly, CD4^+^CD25^−^ responder T cells (10^4^/well), isolated following culture with uRBC, were cultured in the presence of 10^4^ autologous monocytes (also isolated following uRBC stimulation) in 96 well round bottom plates pre-coated with 3 µg/mL anti-CD3 monoclonal antibody (OKT-3, Biolegend). For suppression assays, autologous CD4^+^C25^+^TNFRII^+^ or CD4^+^CD25^+^TNFRII^−^ T cells (isolated following uRBC or pRBC culture) were added at 1∶1 ratio. After 48 hours of culture, cells were pulsed with 1 µCi/well of ^3^[H]-thymidine (Amersham, UK) and incubated for a further 16 h.

### Cell proliferation

100 000 PBMC in culture media (RPMI-1640 (JRH, USA) with 5% heat inactivated human AB serum, 2 mM glutamine, 100 µg/mL streptomycin and 100 U/mL penicillin (all Sigma-Aldrich, USA) were incubated for 5 days in the presence of 10 µg/mL of purified protein derivate (PPD) from *Mycobaterium tuberculosis* (Statens Serum Institute, Denmark), pulsed with 1 µCi of ^3^[H]-thymidine (Amersham, UK) and then incubated for a further 16 h.

### Cytokine assays

The BD CBA array (BD Biosciences, USA) was used according to the manufacturer's instructions to measure the concentrations of IL-10 and TNF in lithium heparin (LiH) plasma and culture supernatant. Soluble TNFRII in LiH plasma was quantitated by ELISA (R&D Systems, Inc. USA) according to the manufacturer's recommendations.

### Statistical analysis

All statistical analyses used GraphPad Prism 5 (Graphpad Software Inc., San Diego, USA). The Kruskal-Wallis test or Mann-Whitney U test compared data among different groups. Spearman rank test was used for correlation analyses. Mixed-effects REML regression was used for analysis of in vitro time course experiments. The Wilcoxon signed rank test was used for analysis of longitudinal data. A significance level of p<0.05 was considered statistically significant.
